# Electronic tongue for determining the limit of detection of human pathogenic bacteria

**DOI:** 10.5599/admet.1650

**Published:** 2023-02-17

**Authors:** Aya Abu Rumaila, Basima Abu Rumaila, Wafa Masoud, Antonio Ruiz-Canales, Nawaf Abu-Khalaf

**Affiliations:** 1 Department of Agricultural Biotechnology, Faculty of Agricultural Sciences and Technology, Palestine Technical University-Kadoorie (PTUK), P.O. Box 7, Jaffa Street, Tulkarm, Palestine; 2 Department of Engineering, School of Engineering of Orihuela (EPSO), Miguel Hernández University (UMH), Carretera de Beniel, km 3.2, 03312 Orihuela, Alicante, Spain

**Keywords:** Electronic tongue, food-borne pathogens, multivariate data analysis, principal component analysis, partial least squares

## Abstract

The Electronic tongue (ET) has been used as a diagnostic technique in the medical sector. It is composed of a multisensor array set with high cross-sensitivity and low selectivity characteristics. The research investigated using Astree II Alpha MOS ET to determine the limit of early detection and diagnosis of food-borne human pathogenic bacteria and to recognize unknown bacterial samples relying on pre-stored models. *Staphylococcus aureus* (ATCC 25923) and *Escherichia coli* (ATCC25922) were proliferated in nutrient broth (NB) medium with original inoculum (approximately 107*10^5^ CFU/mL). They were diluted up to 10^-14^ and the dilutions ranging from 10^-14^ to 10^-4^ were measured using ET. The partial least square (PLS) regression model detected the limit of detection (LOD) of the concentration that was monitored to grow the bacteria with different incubation periods (from 4 to 24 h). The measured data were analysed by principal component analysis (PCA) and followed by projecting unknown bacterial samples (at specific concentrations and time of incubation) to examine the recognition ability of the ET. Astree II ET was able to track bacterial proliferation and metabolic changes in the media at very low concentrations (between the dilutions 10^-11^ and 10^-10^ for both bacteria). *S.aureus* was detected after 6 h incubation period and between 6 and 8 h for *E.coli*. After creating the strains’ models, ET was also able to classify unknown samples according to their foot-printing characteristics in the media (*S.aureus*, *E.coli* or neither of them). The results considered ET a powerful potentiometric tool for the early identification of food-borne microorganisms in their native state within a complex system to save patients’ lives.

## Introduction

Worldwide, food-borne diseases influence public health and cause dangerous diseases. A few pathogenic bacteria are adequate to initiate infection and cause potential damage to the human host system. Patients can be treated for dangerous bacterial diseases after an accurate and early diagnosis of the infection, which requires combining signs and symptoms with precise diagnostic tests to give suitable treatment and avoid unnecessary antibiotics [1,2]. Therefore, finding suitable detecting approaches and developing new and fast methods is important for health and safety. Colony count, enzyme-linked immunosorbent assay (ELISA), electrophoresis, polymerase chain reaction (PCR), biosensors and others have all been employed for the detection of these pathogens [3,4].

The bacterial normal diagnostic process includes culturing, colony counting and phenotypic characteristics. This usually requires 24 to 48 h to grow the pathogen and obtain a pure culture for further antibiotics testing. Moreover, the sensitive and available diagnostic methods (ELISA, PCR nucleic acid detection, antigen testing and surface recognition) are expensive, time-consuming and require a high sophistication level and complex sample preparation [5,6].

Consequently, ultrasensitive, advanced, new methods are required to improve the detecting capability of a few or even a single pathogenic bacterial species in the target samples (such as water, food, blood or biological tissues) [7]. Chemical and biological sensor technologies have recently become popular analytical tools for complex liquid analysis [8-10]. Human smell and taste sensing have been mimicked by the electronic nose (EN) and the electronic tongue (ET) devices (gas and liquid sensors, respectively) and their communication with the human brain [8,11-17]. Liquids and complex solutions can be analyzed using ET systems. They are based on an array of multisensor schemes having pronounced cross-sensitivity and low selectivity characteristics [18-20]. Signals obtained from sensors and liquids are processed with multivariate data analysis (MVDA) techniques, such as principle component analysis (PCA), partial least square (PLS), soft independent model class analogy (SIMCA) and discrimination function analysis (DFA,) allowing for obtaining qualitative and quantitative information on the analyzed samples and creating models from the gathered data [15,18,21-25].

Using ET in the medical analysis is promising to have rapid bacterial detection and shortening the detecting period as much as possible for many physicians, medical laboratories, and even patients as it is an alternative, rapid, reliable and highly sensitive system [26-28].

The limit of detection (LOD) defines the lowest concentration of a variable in a sample that can be constantly detected by a particular measurement process at a specified level of confidence without the necessity of being quantitated as an exact value [29,30].

This research aims to evaluate and/or determine the limit for early detection (LOD) for both the number of colony-forming units (CFU) and incubation or growing periods of food-borne human pathogenic bacteria using ET and multivariate data analysis. Also, to identify unknown bacterial samples relying on a pre-stored bacterial model.

## Experimental

Two bacterial isolates (*Escherichia coli* (ATCC25922) and *Staphylococcus aureus* (ATCC 25923)) obtained from the American Type Culture Collection (ATCC) were cultivated on nutrient agar (NA) medium. NA medium was prepared by dissolving 23 g of NA powder in 1 L distilled water (DW) completely with heating, sterilized at 121 °C and 15 psi for 15 min autoclaving program. The purified medium was cooled and poured into 9 cm Petri dishes under aseptic conditions on a microbiological safety cabinet (MN 120). It was then used for culturing the bacteria.

The plate count was applied for the viable bacterial count. Three fresh well-isolated colonies from NA culture medium were suspended in 1 ml sterile nutrient broth (NB) medium, homogenized using a vortex, and 0.1 ml of stock was serially diluted in 0.9 ml NB tenfolds. This was followed by culturing 0.1 ml of each dilution on NA medium spread with glass hockey sticks and incubating at 37 °C for 24 h. Well-isolated colonies were counted and those within the average of 25-250 CFU were recorded for applying the following equation:

CFU/mL = number of colonies dilution factor / volume of the culture plate

The process was repeated three times for the average count.

Bacterial DNA isolation was applied using TRIzol reagent manual (TRI reagent) (Cat. # T942) (Invitrogen, Thermo Fisher Scientific, US). Three fresh well-isolated colonies from fresh NA culture media were homogenized using vortex in 1 mL of TRI reagent in 1.5 mL microfuge tubes. After that, 200 μL of absolute cold chloroform was added to the suspension, shaken vigorously for 15 sec, and left to stand for 15 min at room temperature. The resulting mixture was centrifuged for 10 min at 11573 rpm at 4 °C to give three phases: colourless upper phase (RNA), interphase (DNA), and red organic phase (protein lower phase). At this point, 300 μL of cold 100 % ethanol was added after removing and discarding the aqueous overlying phase. Tubes were inverted a few times to be mixed and let to stand for 3 min at room temperature, then centrifuged at 4730 rpm for 5 min at 4 °C. The resulting supernatant was removed to be discarded and 1 mL of cold 0.1 M trisodium-citrate in 10 % ethanol solution was used for washing the remaining DNA pellets (twice). Tubes were allowed to stand for 30 min with occasional mixing, centrifuged at 4730 rpm for 5 min at 4 °C, and the resulting pellets were suspended with 1.5 mL of 75 % cold ethanol and allowed to stand for 20 min at room temperature. Later, tubes were centrifuged at 4730 rpm for 5 min at 4 °C discarding the resulting supernatant. In the end, under the vacuum hood, pellets were dried for 10 min, dissolved in 50 μL of TE buffer (add 10.8 g Tris and 5.5 g boric acid in 900 ml distilled water, then add 4 ml 0.5 M Na_2_EDTA (pH 8.0), then adjust the volume to 1 L), and stored at -20 °C for further use.

PCR amplification for the templates was done using a universal 16S bacterial primer set (forward 27F (AGATTTGATCTGGCTCAG)) and reverse primers 1492R (TACGGTTACCTTGTTACGACTT)). The primers were dissolved in sterilized distilled DNase-free water to have a final concentration of 100 μM and stored at -20 °C. PCR amplification mixture was done using Go taq green 2X PCR master mix with 3 mm MgCl_2_ (Cat. # AF9PIM7120418M712). 25 μL PCR reaction mixture contained 12.5 μL of 2X ready mix PCR master mix (75 mM Tris-HCl, 20 mM (NH_4_)_2_SO_4_, 0.625 U Thermo prime taq DNA polymerase, 0.2 mM of each dNTPs, 1.5 mM MgCl_2_), 0.5 μL of 50 mM MgCl_2_, 0.125 μL of 100 μM forward primer, 0.125 μL of 100 μM reverse primer, 10.75 μL of free DNase water and 1 μL of DNA template.

VertiTM 96 well thermal cycler (Cat. #: 4375786) (Applied Biosystems company, California, USA) was used to perform a PCR amplification program. The program started with an initial 94 °C cycle for 3 min, followed by 35 cycles of 45-sec denaturation cycle at 94 °C, 50 sec of 51 °C, and 1 min at 72 °C, and then 7 min of the final cycle at 72 °C.

The PCR procedure was duplicated for each isolate to guarantee the reproducibility of the amplified DNA fragments. A blank negative control sample was also run. To separate the total extracted bacterial DNA, a 0.8 % agarose electrophoresis gel was used. Meanwhile, 2 % agarose electrophoresis gel was prepared to separate PCR products. The gel was prepared by dissolving 2 g of agarose powder completely in 100 mL of 1X TBE buffer with heating using the microwave. The mixture was cooled to 60 °C. After that, 4 μL of 1000X Gel Red DNA stain (Cat. #41003) (Med Chem Express, USA) was added and stirred. The suspension was then powered and allowed to solidify in a (10 x 10) tray with 13 wells comp. After submerging the gel in 1 X TBE buffer, 5 μL of PCR products were loaded and the device was run for 2 h at 70 volts.

A 10000X Gel Red DNA stain and UV-illuminator were used to visualize DNA fragments and SynGene gene tool system (Synoptics Ltd., Cambridge C, UK) was used to document it using image acquisition and documentation. For estimating DNA fragments size, a DNA ready-to-use (RTU) ladder (Cat. # DM001-R500) of 100 bp was used as a molecular marker. Finally, PCR products were stored and sent for sequencing. The obtained bacterial sequences were aligned using the universal BLAST program (National Center for Biotechnology Information, Maryland, USA).

Meanwhile, for ET measurements, a liquid taste analyzer Astree II ET (Alpha MOS Company, Toulouse, France) was used. That is composed of seven sensor arrays (CA, JB, HA, ZZ, BB, JE and GA) with an Ag/AgCl reference electrode. Five testing rounds of bacterial samples were measured on ET. The first round was for determining the limit of detection (LOD) (limited CFU) for *E.coli* samples that ET can detect after 24 h incubation period. The second was for determining the least incubation time for *E.coli* that ET can detect after cultivating the detected least CFU (the same two rounds were applied for *S.aureus*). The final fifth round was done to test ET capability to recognize unknown bacterial samples of *E.coli*, *S.aureus*, and others (*S.agalactiae* and *P.aeruginosa*) that were grown at the least incubation time and CFU.

In each round, 11 bacterial samples with a Nutrient broth (NB) media sample (control) were tested in triplicate. NB was prepared by dissolving a complete weight of 13 g NB powder in 1 L DW by heating, suspended in 250 mL Erlenmeyer flasks, each containing 100 mL of the suspension that was labelled and sealed with aluminium foil for autoclaving at 121 °C and 15 psi for 15 min, and left to cool. The overall action was also done at aseptic conditions. Bacterial proliferation was done by cultivating three fresh colonies (approx. 107h10^5^ CFU/mL) of pure cultured bacteria in 100 ml NB media. The dilution test was applied by serially diluting 1 ml of stock in 99 ml of sterilized NB media up to 14 folds. Flasks were then incubated at 37 °C with shaking at 150 rpm for 24 h (the samples with dilutions 10^-14^ to 10^-4^ were analyzed using ET). Meanwhile, the growth period test was applied by inoculating the media with the determined least concentration CFU (approx. 88 10^-9^ CFU/mL) of each bacterial type that was then incubated at 37 °C with shaking at 150 rpm for different periods (4, 6, 8, 10, 12, 14, 16, 18, 20, 22 and 24 h). In the fifth final round, bacterial samples of *E.coli, S.aureus, S.agalactiae* and *P.aeruginosa* with NB as a control sample were tested. Those samples were measured at 10^-9^ CFU concentration after 10, 12 and 14 h of inoculation to identify unknown bacterial samples relying on pre-stored bacterial data and if it can recognize them from other types of bacteria.

To create the sequence on ET a two parts labelling was applied, where the first part has the bacterial name (i.e. *E.coli, Staph*, *UnEc, UnSa, UnPs, UnSr and UnNB*), the other for the concentration (i.e., -04 to -14 or NB) and/or incubation period (i.e., 04h to 24h or NB) (Table 1).

Before ET testing, bacterial samples were filtered using a white cheesecloth to obtain approximately 80 mL of each broth to be placed on ET’s 16-position autosampler, with an automatic stirrer, after creating the sequence. Samples were separated by four water-cleaning samples for cleaning ET sensors after each test.

After each measurement, the data from each sensor was collected in a folder categorized by bacterial sequence for each round after creating a library of the experiment.

The collected raw data from analyzed sensors were exported to Unscrambler X (version 10.3, Camo Software AS, Oslo, Norway), where the signals of each sensor were numerically analyzed and normalized to values to be categorized using PLS and PCA.

## Results and Discussion

### Bacterial experiment

#### Bacterial colony forming unit (CFU) counting

**Figure 1.**
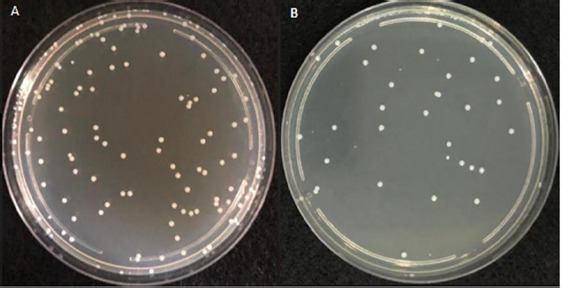
Plated bacterial dilution of 88*10^-9^ CFU/mL with well-separated and countable colonies. A: plate with *E.coli*, B: plate with *S.aureus*.

Figure 1 represents the plated bacterial dilution (approximately 88*10^-9^ CFU/mL) with well-separated and countable colonies of 25-250 CFU, considered for the ET LOD testing procedure.

#### Bacterial DNA isolation and PCR

**Figure 2.**
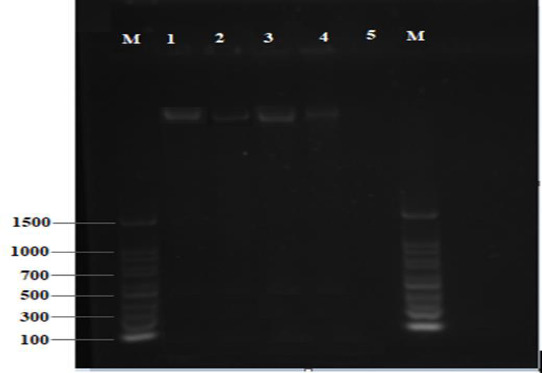
Gel electrophoresis documentation of bacterial total DNA isolation using TRI reagent method. Where lanes 1 and 2 represent *E.coli* samples, 3 and 4 represent *S.aureus* samples, 5 is a negative control. M=100 bp ladder as a molecular size marker.

**Figure 3.**
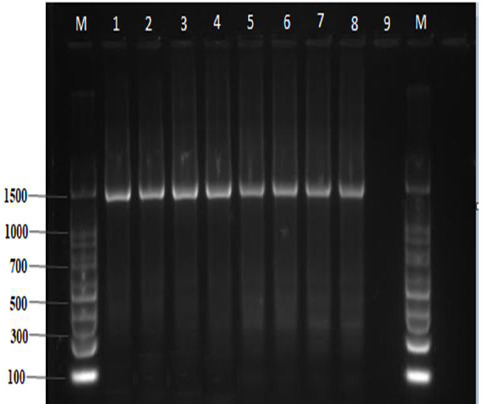
Gel electrophoresis documentation of bacterial 16S rRNA amplification in eight bacterial isolates using primer 27F and 1492R. 1-4 represents *E.coli* samples, 4-8 represents *S.aureus* samples and 9 is a negative control M=100 bp ladder as a molecular size marker.

The total DNA extracted from four bacterial samples using TRI reagent method is shown in Figure 2. PCR amplification of DNA templates using a universal 16S bacterial primer set (27F and 1492R) resulted in 1500 pb bands used for the sequencing process (Figure 3).

#### Sequence identification

**Figure 4.**
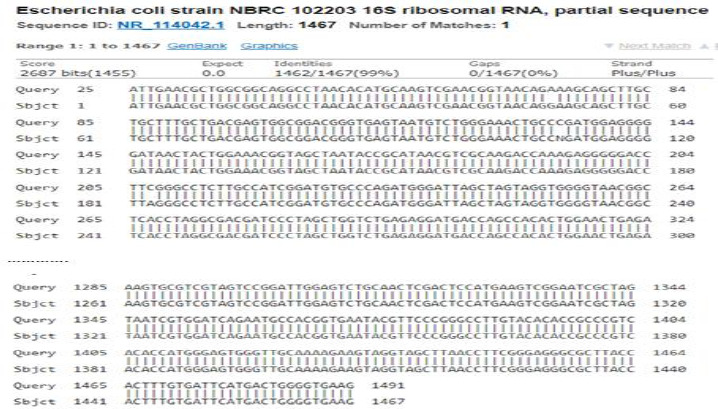
BLASTn alignment for *E.coli* sequenced 16S ribosomal RNA with 99 % identity to Escherichia coli strain NBRC 102203.

**Figure 5.**
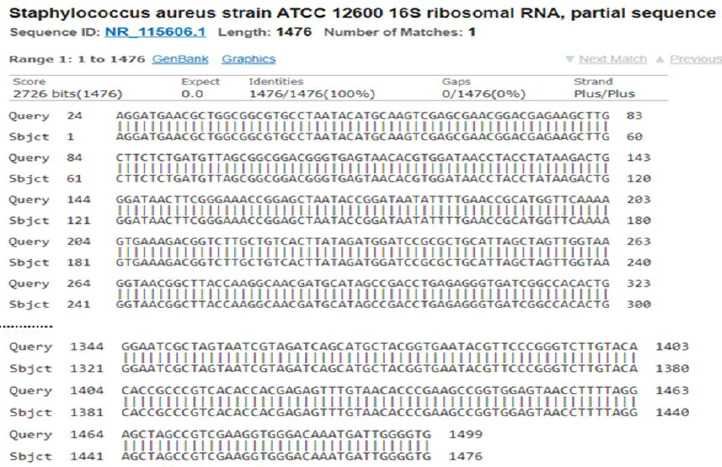
BLASTn alignment of *S.aureus* sequenced 16S ribosomal RNA with 100 % identity to Staphylococcus aureus ATCC 12600.

BLASTn alignment of the 16S rRNA gene sequences of *E.coli* and *S.aureus* bacterial samples are shown in Figure 4 and Figure 5, respectively. It shows obtained sequence homology of 99 % for *E.coli* to strain NBRC 102203 and 100 % for *S.aureus* to strain ATCC 12600.

### ET data analysis

#### LOD test of bacterial concentration

**Figure 6.**
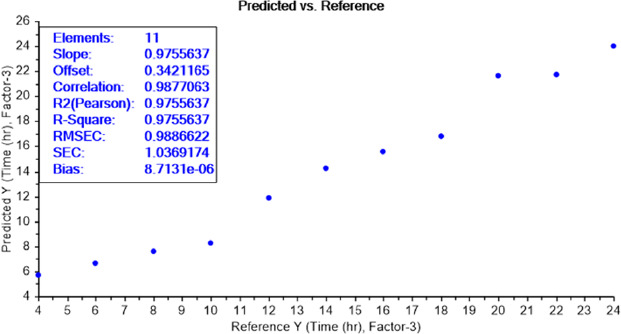
PLS recognition model for *E.coli* LOD of different dilutions ranged from 10^-14^ to 10^-4^. ET can sense the presence of bacteria, in NB media, between dilutions 10-^11^ and 10^-10^.

**Figure 7.**
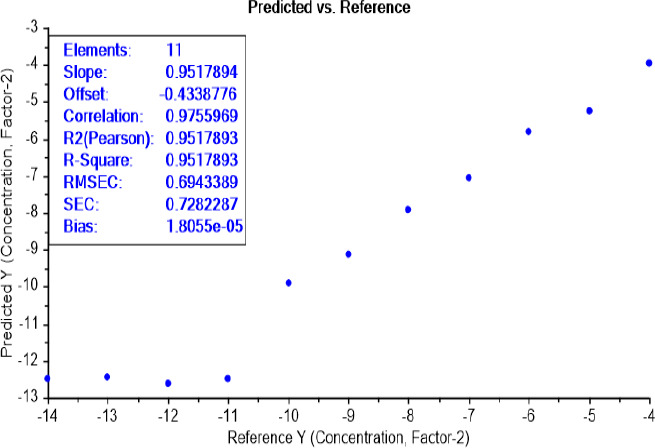
PLS recognition model for *S.aureus* LOD of different dilutions ranged from 10^-14^ to 10^-4^. ET can sense the presence of bacteria, in NB media, between dilutions 10^-11^ and 10^-10^.

The calibration curve of the PLS recognition model, for determining the limit of detection (LOD) test of bacterial concentration, has identified the presence of bacteria between the dilutions 10^-11^ and 10^-10^ for both bacteria *E.coli* (Figure 6) and *S.aureus* (Figure 7).

#### LOD test of bacterial earliest incubation period

**Figure 8.**
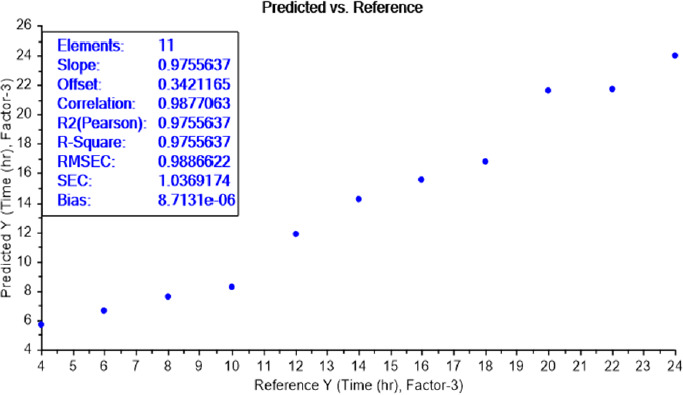
PLS recognition model for *E.coli* LOD of different incubation periods ranged from 4 to 24 h. ET can sense the presence of bacteria, in NB media, between incubation periods 6 and 8 h.

**Figure 9.**
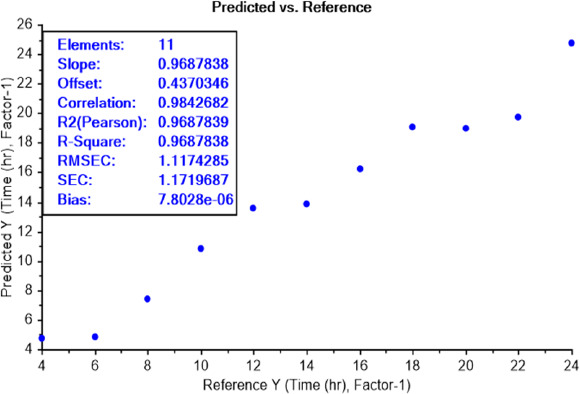
PLS recognition model for *S.aureus* LOD of different incubation periods ranged from 4 to 24 h. ET can sense the presence of bacteria, in NB media, at an incubation period of 6 h.

The calibration curve of the PLS recognition model for determining the LOD test of bacterial earliest incubation period after determining the concentration LOD (10^-9^) identified that ET can sense the presence of *E.coli*, in NB media, between 6 and 8 h of incubation (Figure 8) and *S.aureus* after 6 h of incubation (Figure 9). The results are summarized in Table 2.

#### ET classification test

**Figure 10.**
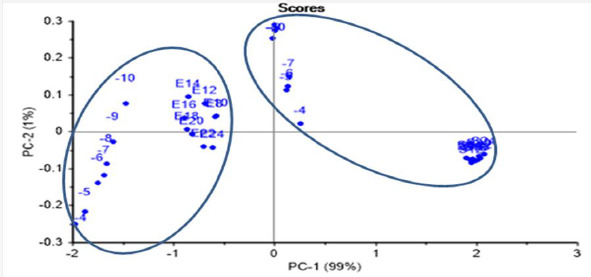
PCA scores plot for both bacterial data at the recognized LOD tests (dilution greater than 10^-10^ and growth time greater than 8 h). E: *E.coli*, S: *S.aureus*.

ET was able to identify two well-separated groups of *E.coli* and *S.aureus* in the same PCA scores plot, after joining the data for both recognized LOD tests (dilution greater than 10^-10^ and growth time greater than 8 h) in the same PCA score plot (Figure 10). The scoring model had 99 % PC-1 recognition power.

#### ET projection model

A PCA model for *E.coli* and *S.aureus* were created using the resulting data for the projection test, where unknown samples of *E.coli* and *S.aureus* were incubated with a dilution of 10^-9^ for 10, 12 and 14 h, and *S.agalactiae* and *P.aeruginosa* as gram-positive and gram-negative bacteria were also incubated with a dilution of 10^-9^ and for 14 h in order to test the created models and to prove ET’s ability to recognise between different bacterial samples.

**Figure 11.**
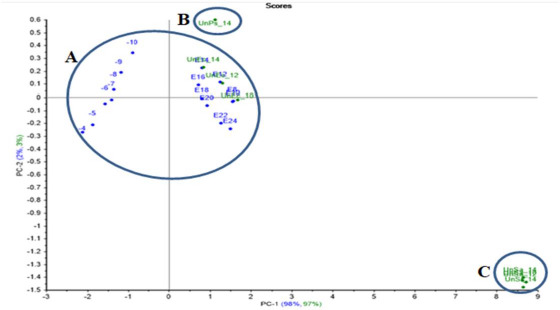
*E.coli* PCA projection model with projected unknown samples. A: a group of *E.coli*’s created data with projected unknown *E.coli*s samples (incubated with a dilution of 10^-9^ and periods at 10, 12 and 14 h), B: projected unknown *P.aeruginosa* (incubated with a dilution of 10^-9^ and 14 h), C: projected unknown *S.aureus* and *S.agalactiae* that incubated with a dilution of 10^-9^ and periods at 10, 12 and 14 h.

*E.coli* PCA projection model projected unknown *E.coli* samples close enough to the created models’ data. Meanwhile, the unknown *P.aeruginosa* was out of the group, as well as the projected unknown *S.aureus* and *S.agalactiae*, which were far away from the model group (Figure 11).

**Figure 12.**
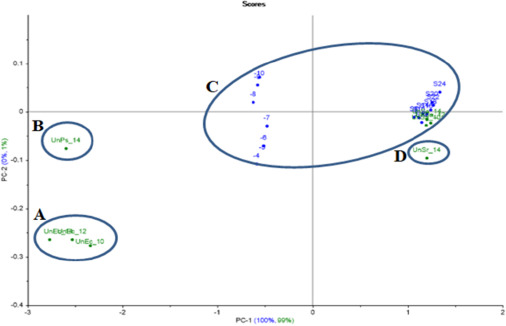
*S.aureus* PCA projection model with projected unknown samples. A: projected unknown *E.coli* samples (incubated with a dilution of 10^-9^ and periods at 10, 12 and 14 h), B: projected unknown *P.aeruginosa* (incubated with a dilution of 10^-9^ and 14 h), C: a group of all *S.aureus*’s created data with projected unknown *S.aureus* samples (incubated with a dilution of 10^-9^ and periods at 10, 12 and 14 h), D: projected *S.agalactiae* (incubated with a dilution of 10^-9^ and 14 h).

*S.aureus* PCA projection model projected samples of unknown *S.aureus* inside the model’s created group. Meanwhile, the unknown *S.agalactiae* was out of the group (at a distance), as well as the projected unknown *E.coli* and *P.aeruginosa*, which were far away from the model group (Figure 12).

The 99 % homology for *E. coli* may be due to mutations throughout the subsequent culturing or the sequencing process. It can also be attributed that *E. coli* used in this study is a different strain from strain NBRC 102203.

The ET was able to classify the two types of bacteria according to their gram-negative and gram-positive strains (i.e., *E.coli* and *S.aureus*). Moreover, ET could sense the difference between the same strains (i.e., *E.coli* and *P.aeruginosa* as gram-negative, and *S.aureus* and *S.agalactiae* as gram-positive). This can be due to bacteria’s different characteristics.

## Conclusions

Astree II ET was an efficient technique for tracking bacterial growth and following their metabolic changes in NB media. It was able to create a categorization model that is specific for some strains of microorganisms. Moreover, ET was able to detect these food-borne bacterial strains just a few hours after inoculation up to only 8 h and even 6 h in some strains such as *S.aureus*. ET’s sensitivity was also confirmed for identifying microorganisms’ proliferation even with a very low concentration of an original inoculum (such as a dilution factor up to 10^-10^).

According to these statements, ET can be considered a powerful tool for early identification and fast classification of harmful food-borne microorganisms by creating other subsequent steps to create microorganisms’ models and save patients’ lives. In the long term, this will open a wide door for using these sensors as an alternative assessment and fast monitoring technique in industrial, categorizing, fermentable and other applications.

ET ease of use in tracking microorganism footprints coupled with distinguishing these microorganisms in the native state (in vitro assessment) and being contained in a complex system is important. However, combining ET with other technologies can provide a powerful combination in a wide range of applications.

Further studies should be carried out to monitor sensors' temperature dependence and charge transfer affected by the adsorption of solution components. Also, enlarging the specified foot-printing databases of microorganisms that needs the first step of full work.

## Figures and Tables

**Table 1.** The ET five experiments rounds and the labelling for each tested sample

Round No.	Sample No.	Bacterial type	Dilution factor	The incubation period, h	ET code	Goal of the experiment
Round 1	1	*E.coli*	10^-4^	24	E.coli_-04	*E.coli* concentration LOD test
	2	*E.coli*	10^-5^	24	E.coli_-05	
	3	*E.coli*	10^-6^	24	E.coli_-06	
	4	*E.coli*	10^-7^	24	E.coli_-07	
	5	*E.coli*	10^-8^	24	E.coli_-08	
	6	*E.coli*	10^-9^	24	E.coli_-09	
	7	*E.coli*	10^-10^	24	E.coli_-10	
	8	*E.coli*	10^-11^	24	E.coli_-11	
	9	*E.coli*	10^-12^	24	E.coli_-12	
	10	*E.coli*	10^-13^	24	E.coli_-13	
	11	*E.coli*	10^-14^	24	E.coli_-14	
	12	*--------*	-------	24	E.coli_NB	
Round 2	1	*E.coli*	10^-9^	4	E.coli_04h	*E.coli* LOD for incubation periods test
	2	*E.coli*	10^-9^	6	E.coli_06h	
	3	*E.coli*	10^-9^	8	E.coli_08h	
	4	*E.coli*	10^-9^	10	E.coli_10h	
	5	*E.coli*	10^-9^	12	E.coli_12h	
	6	*E.coli*	10^-9^	14	E.coli_14h	
	7	*E.coli*	10^-9^	16	E.coli_16h	
	8	*E.coli*	10^-9^	18	E.coli_18h	
	9	*E.coli*	10^-9^	20	E.coli_20h	
	10	*E.coli*	10^-9^	22	E.coli_22h	
	11	*E.coli*	10^-9^	24	E.coli_24h	
	12	*--------*	-------	24	E.coli_NB	
Round 3	1	*S.aureus*	10^-4^	24	Staph_-04	*S.aureus* concentration LOD test
	2	*S.aureus*	10^-5^	24	Staph_-05	
	3	*S.aureus*	10^-6^	24	Staph_-06	
	4	*S.aureus*	10^-7^	24	Staph_-07	
	5	*S.aureus*	10^-8^	24	Staph_-08	
	6	*S.aureus*	10^-9^	24	Staph_-09	
	7	*S.aureus*	10^-10^	24	Staph_-10	
	8	*S.aureus*	10^-11^	24	Staph_-11	
	9	*S.aureus*	10^-12^	24	Staph_-12	
	10	*S.aureus*	10^-13^	24	Staph_-13	
	11	*S.aureus*	10^-14^	24	Staph_-14	
	12	*--------*	-------	24	Staph_NB	
Round 4	1	*S.aureus*	10^-9^	4	Staph_04h	*S.aureus* LOD for incubation periods test
	2	*S.aureus*	10^-9^	6	Staph_06h	
	3	*S.aureus*	10^-9^	8	Staph_08h	
	4	*S.aureus*	10^-9^	10	Staph_10h	
	5	*S.aureus*	10^-9^	12	Staph_12h	
	6	*S.aureus*	10^-9^	14	Staph_14h	
	7	*S.aureus*	10^-9^	16	Staph_16h	
	8	*S.aureus*	10^-9^	18	Staph_18h	
	9	*S.aureus*	10^-9^	20	Staph_20h	
	10	*S.aureus*	10^-9^	22	Staph_22h	
	11	*S.aureus*	10^-9^	24	Staph_24h	
	12	--------	-------	24	Staph_NB	
Round 5	1	*E.coli*	10^-9^	10	UnEc_10	Identify unknown bacterial samples relaying on pre-stored bacterial model
	2	*E.coli*	10^-9^	12	UnEc_12	
	3	*E.coli*	10^-9^	14	UnEc_14	
	4	*S.aureus*	10^-9^	10	UnSa_10	
	5	*S.aureus*	10^-9^	12	UnSa_12	
	6	*S.aureus*	10^-9^	14	UnSa_14	
	8	*P.aeruginosa*	10^-9^	14	UnPs_14	
	10	*S.agalactiae*	10^-9^	14	UnSr_14	
	11	--------	--------	12	UnNB_01	
	12	--------	--------	14	UnNB_01	

**Table 2.** Limit of detection (LOD) results for *S.aureus* and *E.coli*.

Bacterial type	LOD of Concentration	LOD of the incubation period
*S.aureus*	Between 10^-11^ and 10^-10^	After 6 h
*E.coli*	Between 10^-11^ and 10^-10^	Between 6 and 8 h
